# Short-term safety and cosmetic outcomes of endoscopic direct-to-implant breast reconstruction and simultaneous contralateral breast augmentation for breast cancer: a prospective analysis of 33 patients

**DOI:** 10.1186/s12957-023-03089-4

**Published:** 2023-07-10

**Authors:** Mengxue Qiu, Huanzuo Yang, Jiao Zhou, Yu Feng, Xinran Liu, Qing Zhang, Zhenggui Du

**Affiliations:** 1 Department of General Surgery, West China Hospital, Sichuan University, 37 Guoxue Street, WuHou District, Chengdu, 610041 China; 2 Breast Center, West China Hospital, Sichuan University, 37 Guoxue Street, WuHou District, Chengdu, 610041 China

**Keywords:** Breast cancer, Endoscopic, Direct-to-implant, Breast reconstruction, Breast augmentation

## Abstract

**Background:**

For patients with small breasts, breast-conserving surgery (BCS) and unilateral nipple-/skin-sparing mastectomy (N/SSM) with breast reconstruction may result in visible breast deformities or asymmetry, and contralateral breast augmentation often require a two-staged operation. We propose a novel endoscopic technique, direct-to-implant breast reconstruction and simultaneous contralateral breast augmentation (DTI-BR-SCBA), and report its short-term safety and cosmetic outcomes.

**Methods:**

In this prospective study, patients with early breast cancer who underwent endoscopic DTI-BR-SCBA between November 2020 and August 2022 were followed for more than 3 months to analysed short-term postoperative safety (complications and oncological safety) and cosmetic outcomes (doctor-assessed results by Ueda scale and patient-reported results by Breast-Q scale).

**Results:**

A total of 33 patients, including 30 treated with endoscopic prepectoral DTI-BR-SCBA, 1 with endoscopic dual-plane DTI-BR-SCBA and 2 with endoscopic subpectoral DTI-BR-SCBA, were analysed. The mean age was 39.7 ± 6.7 years. The mean operation time was 165.1 ± 36.1 min. The overall surgical complication rate was 18.2%. All complications were minor, including haemorrhage (3.0%), cured by compression haemostasis, surgical site infection (9.1%), cured by oral antibiotics, and self-healing nipple-areolar complex ischaemia (6.1%). Furthermore, rippling and implant edge visibility occurred in 6.2% of them. The outcome was graded as “Excellent” and “Good” in 87.9% and 12.1% of patients in the doctor cosmetic assessment, respectively, and patient satisfaction with breasts was significantly improved (55.0 ± 9.5 vs. 58.8 ± 7.9, *P* = 0.046).

**Conclusions:**

The novel endoscopic DTI-BR-SCBA method may be an ideal alternative for patients with small breasts because it can improve cosmetic results with a relatively low complications rate, which makes it worthy of clinical promotion.

**Supplementary Information:**

The online version contains supplementary material available at 10.1186/s12957-023-03089-4.

## Introduction

Breast cancer is the most common malignancy in women worldwide [1–3] and is a serious threat to women's physical and mental health. For patients with small breasts, breast conserving surgery (BCS) and unilateral breast construction may not be the ideal choice, and unilateral breast reconstruction combined with contralateral breast augmentation can create a more symmetrical appearance.

Obvious incisions on the breasts cannot be avoided in traditional nipple-/skin-sparing mastectomy (N/SSM), which not only affects aesthetics but also increases the risk of incision-related complications due to damage to the flap blood supply [4]. Given that high skin tension after implant placement can easily lead to flap rupture, traditional breast reconstruction following N/SSM and contralateral breast augmentation usually requires two stages, namely a first stage for expander placement and a second stage for implant replacement with simultaneous contralateral breast augmentation, which increases the physical, mental and financial burdens on patients.

Endoscopic or robotic surgery, as opposed to open procedures, is advantageous in that it minimizes surgical scarring [5–7] and the risk of direct-to-implant breast reconstruction (DTI-BR); however, the difficulty of the operation, long surgery time and high cost limit its regular application in patients [8–11]. In view of the existing dilemma, our team has made breakthroughs in endoscopic N/SSM [12–15]. The innovative reverse sequence dissection can build enough space and prevent mutual instrument interference inside the cavity, and the use of “HUAXI Hole 1” can reduce the difficulty of removing the mammary gland in the lower-inner quadrant, thus improving surgical operability and shortening surgical time. Non-scarring of breasts can reduce the risk of wound dehiscence and implant loss in immediate breast reconstruction, and avoid the trauma and financial impact of two-staged surgery. Our preliminary study results showed that endoscopic N/SSM and DTI-BR had reliable safety and cosmetic outcomes [12, 15, 16] and could be performed at a 24-h admission centre [17].

In this study, we proposed a novel surgical technique, transaxillary endoscopic direct-to-implant breast reconstruction and simultaneous contralateral breast augmentation (DTI-BR-SCBA), for breast cancer patients with small breasts (with no or mild-to-moderate breast ptosis), and prospectively analysed the short-term safety and cosmetic outcomes in 33 patients from the West China Hospital of Sichuan University.

## Methods

### Patients

The novel endoscopic technique can be performed in any patient aged ≥ 18 years who has small breasts (with no or mild-to-moderate breast ptosis) or self-dissatisfied breasts, hopes to improve the appearance of the breasts and meets the indications for N/SSM, including patients with early breast cancer (carcinoma in situ, stage I or II cancer) with contraindications to or who are unwilling to undergo BCS and radiotherapy following BCS, with a tumour size less than 5 cm initially or after neoadjuvant chemotherapy, and with no evidence of multiple lymph node metastases (cN0 and cN1). Patients with skin or chest wall invasion, severe comorbid conditions and pregnancy or lactation were excluded from this study.

In this study, 33 patients who underwent endoscopic DTI-BR-SCBA at the West China Hospital between November 2020 and August 2022 after verification of indications and exclusion of contraindications were recruited for this prospective study. The study was approved by the Ethics Committee on Biomedical Research of the West China Hospital of Sichuan University (No. [2021]592) and registered with the Chinese Clinical Trial Registry (No. ChiCTR2100047081). All patients signed informed consent forms and agreed to the publication of their photos or videos.

### Surgical procedure

#### Markings and contralateral breast augmentation

**Fig. 1:**
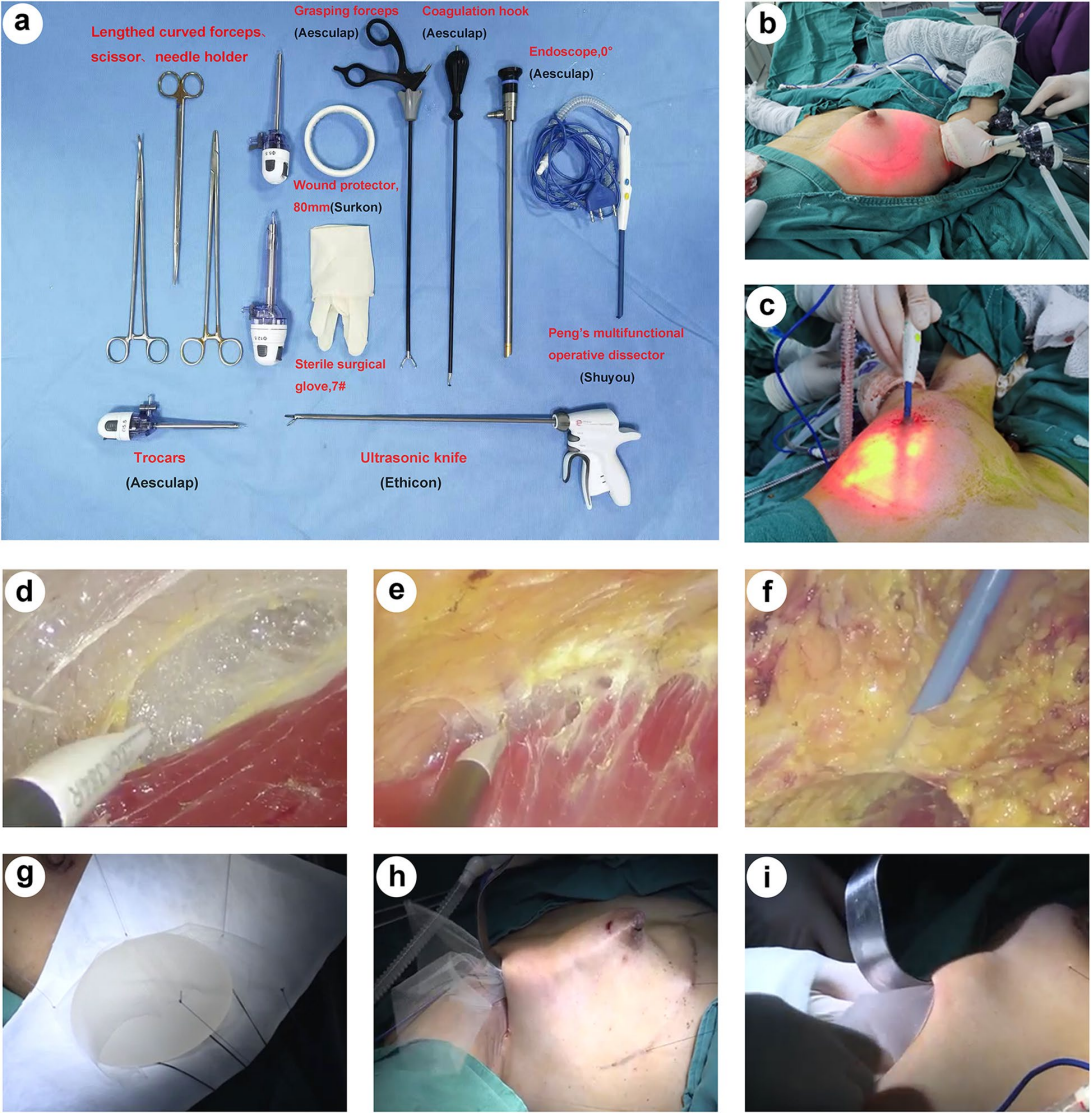
Photos of surgical instruments and procedures. **a** Commonly used instruments in endoscopic nipple-/skin-sparing mastectomy with direct-to-implant breast reconstruction and simultaneous contralateral breast augmentation. **b** Endoscopic contralateral breast augmentation and axillary operating device. **c** Subcutaneous dissection (inner lower quadrant) using “HUAXI Hole 1”. **d** Subpectoral layer dissection. (I) Retromammary space dissection. **f** Subcutaneous dissection. **g** In vitro display of implant and mesh placement. **h** Parachute mesh placement. **i** Implant placement

Cutaneous markings were drawn preoperatively with the patient in the standing position to mark the anterior midline, bilateral inframammary fold (IMF), tumour site and breast gland boundary. The new IMF was designed according to the patient's desired breast size and we found that moving the new IMF 0.75 cm to 1 cm down for each increase in new breast cup size can avoid excessive fullness of the upper breast caused by the implant. A 3–5-cm incision marking line was made at the subaxillary fold and was completely covered when the upper limbs were at rest. The surgical instruments are shown in Fig. 1a.

To avoid tumour metastasis caused by the operation, contralateral breast augmentation was performed first, followed by ipsilateral N/SSM. Breast augmentation was performed under endoscopy with gas insufflation, and implants on the breast augmentation side and reconstruction side were placed on the same layer (prepectoral or subpectoral), but no mesh was used for breast augmentation [18–20]. There were three breast reconstruction methods in this study. Prepectoral breast reconstruction was a viable option for the majority of patients; however, dual plane breast reconstruction was more suitable for those with thin flaps or ptotic breasts, as it could reduce rippling and improve breast ptosis. For patients with financial difficulties, subpectoral breast reconstruction without mesh might be considered. See the movie in Additional file 11 for the endoscopic prepectoral breast augmentation procedure.

#### Working space creation and NSM

**Fig. 2:**
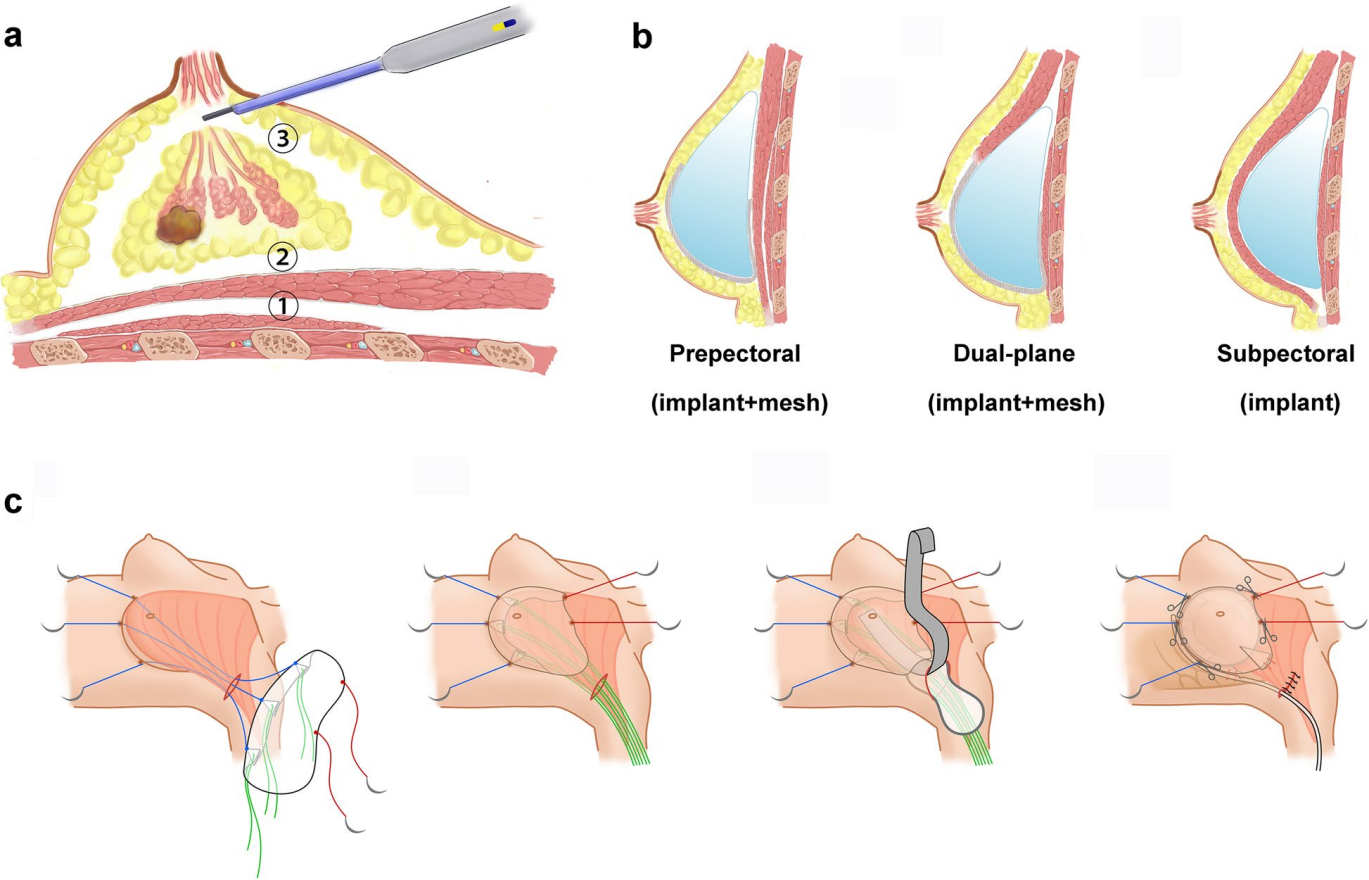
Schematic diagrams of this surgery for endoscopic breast reconstruction. **a** Separation sequence of endoscopic nipple-sparing mastectomy with implant-based reconstruction: subpectoral layer (not required for prepectoral reconstruction) → retromammary layer → subcutaneous layer. **b** Prepectoral, dual-plane and subpectoral breast reconstruction. **c** Implant and mesh placement

Sentinel lymph node biopsy (SLNB) or axillary lymph node dissection (ALND) was performed prior to NSM under direct vision through the axillary incision. Before creating the working space, the subpectoral, retromammary and subcutaneous layers were dissected in 8–10 cm, 5–8 cm and 3–5 cm areas with an electrotome under direct vision. A 60- or 80-mm disposable wound protector was placed through the incision and wrapped using the open end of one sterile surgical glove to seal the wound cavity. Three different fingertips of the glove were cut off and used as channels. Two bladeless trocars were inserted into different fingerholes with threads fixed to create entry sites for the endoscope and grasping forceps or a coagulation hook, while the other fingerhole was for the electrotome. The cavity was filled with CO2 [12 mmHg (1 mm Hg = 0.133 kPa), 20–40 L/min] to maintain patency and sufficient tension (Fig. 1b). The order of layer dissociation in NSM was opposite to the traditional order, as shown in Fig. 2a. First, the coagulation hook or electrotome was used to dissociate the subpectoral layer and form the implant cavity (Fig. 1d). Due to the pressure of the air chamber, the pectoralis major and upper gland will be raised, which can help to further dissociate the tissues downwards to the new IMF. For prepectoral breast reconstruction, there was no need to dissociate the subpectoral layer.

Then, the retromammary space was dissociated under endoscopic vision in the same manner as described above (Fig. 1e). The range of dissection extended downwards to the original IMF in subpectoral breast reconstruction, and at this point, in dual-plane breast reconstruction, the distal end of the pectoralis major was cut off medially up to the fifth intercostal and laterally up to the serratus anterior fascia. For dual-plane and prepectoral reconstruction, additional dissociation down to the new IMF in this layer was needed.

Finally, on the subcutaneous plane, the lower-outer, upper-outer and part of upper-inner quadrants were dissected with an electrotome through the axillary incision under endoscopic vision to reach the root of the nipple. The central gland was dissociated along the areola dermis, and the transected end of the nipple was sent for intraoperative frozen sectioning. The remaining inaccessible quadrants were dissected under endoscopic vision by means of “HUAXI Hole 1”; a 0.2-cm skin incision was created at the superolateral margin of the areola (Fig. 1c, 1f). The whole gland was then excised and removed completely via the axillary incision. See the movie in Additional file 2 the for endoscopic NSM procedure (followed by prepectoral breast reconstruction).

#### Implant and mesh placement

After rinsing the cavity, the implant was placed (combined with mesh placement in prepectoral and dual-plane breast reconstruction), as shown in Fig. 1g–i and Fig. 2b–c. The TiLOOP mesh was folded according to the shape of the inframammary fold with a width of approximately 1.5–2 cm. Three absorbable sutures (pull-down sutures) were sutured on the middle, medial and lateral sides of the folded edge, three silk sutures (return sutures) were parallel to the three downwards stitches and pulled out through the axillary incision to hook the bottom edge of the mesh, and two absorbable sutures (upwards traction sutures) were sewn onto the mesh to drag the surface layer of the patch upwards to avoid folding. Through the axillary incision, three pull-down sutures were sewn out of the skin along the IMF and pulled downwards such that the mesh dropped like a parachute and was fixed by traction stitches. The upper end of the mesh was directly sutured on the cut end of the pectoralis major in dual-plane breast reconstruction. Then, the breast implant was placed into the pocket formed by the mesh (or mesh and pectoralis major muscle). In our experience, the choice of implant size for most patients follows the formula of “reconstruction side implant size (cc) = augmentation side implant size (cc) + excised gland weight (g) × (90–95)%”, but recommendations need to be individualized when applied clinically. A drainage tube was placed into the cavity, and the axillary incision was sutured. See the movie in Additional file 3 for the endoscopic prepectoral breast reconstruction procedure.

### Outcome assessment

The foci of the study were short-term safety and cosmetic results at 3 months after surgery. In terms of safety outcome evaluation, intraoperative adverse events, postoperative complications (surgical and cosmetic), local recurrence and distant metastasis were recorded. The postoperative complications were recorded according to the Clavien-Dindo classification [21] and defined as minor (Clavien-Dindo grades I–II) and major complications (Clavien-Dindo grades ≥ III).

Cosmetic outcomes included doctor-assessed results and patient-reported results. Photographs were taken pre- and postoperatively for cosmetic evaluation by 3 different breast surgeons (not involved in the surgeries) using a scoring system established by the Japanese Breast Cancer Society (hereafter “Ueda scale”) [22], which was used to evaluate breast, nipple-areolar complex (NAC) and inframammary lines to categorize patient outcomes as “Poor”, “Fair”, “Good” or “Excellent”. Patient satisfaction was estimated using the Breast-Q [23], which included psychosocial well-being, sexual well-being, chest well-being and satisfaction with breasts.

### Statistical analysis

Measurement data are expressed as the mean ± standard deviation, and count data are expressed as percentages. SPSS (version 25.0, SPSS, Inc., Chicago, IL) was used for statistical analysis and R (version 4.0.2, R Development Core Team 2020) was used for mapping. The preoperative and 3-month postoperative Breast-Q scores were compared by the paired t test. A *P* value < 0.05 was considered statistically significant.

## Results

### Clinical characteristics and operative data

A total of 33 breast cancer patients who underwent transaxillary endoscopic DTI-BR-SCBA were recruited for this study. The mean age was 39.7 ± 6.7 years. Most of the patients had small breasts before surgery (81.8% ≤ breast cup size A), and 5 patients had grade 1 to 2 breast ptosis. The average duration of the operation was 165.1 ± 36.1 min, including 24.9 ± 11.5 min for axillary management, 50.7 ± 9.1 min for mastectomy, 22.2 ± 4.6 min for breast reconstruction and 20.4 ± 6.1 min for breast augmentation. The weight of the excised glands was 174.5 ± 56.2 g. The mean implant volume for breast reconstruction and augmentation was 320.3 ± 56.7 cc and 179.4 ± 26.5 cc, respectively. All operations were conducted smoothly, without intraoperative complications. The surgery was performed at a 24-h admission centre for 18 patients (54.5%), while 15 patients (45.5%) required inpatient admission, with a mean length of hospital stay of 3.6 ± 3.3 days. The clinical characteristics and operative data are summarized in Table 1.

**Table 1:** Patient characteristics and operative data

		All patients *N* = 33 (%)
Age		39.7 ± 6.7
BMI		20.5 ± 1.8
Smoking		0(0)
Alcohol drinking		4 (12.1)
Comorbidity	No	31 (93.9)
	Hypertension	2 (6.1)
	Diabetes mellitus	0 (0)
	Others	0 (0)
Breast ptosis^a^	Normal	28 (84.8)
	Pseudo	0 (0)
	Grade 1	3 (9.1)
	Grade 2	2 (6.1)
	Grade 3	0 (0)
Preoperative breast cup size^b^	AA	17 (51.5)
	A	10 (30.3)
	B	6 (18.2)
	≥ C	0 (0)
Tumour site	Left breast	15 (45.5)
	Right breast	18 (54.5)
Histology	Ductal carcinoma in situ	9 (27.3)
	Invasive carcinoma	24 (72.7)
Clinical stage	0	10 (30.3)
	I	13 (43.3)
	II	10 (30.3)
Lymph node surgery	SLNB only	25 (75.8)
	SLNB then ALND	4 (12.1)
	ALND	4 (12.1)
Nipple excision		1 (3.0)
Breast reconstruction and augmentation methods	Subpectoral	2 (6.1)
	Dual-plane	1 (3.0)
	Prepectoral	30 (90.9)
Operation time (minutes)		165.1 ± 36.1 (113–268)
Anaesthesia time (minutes)		238.7 ± 47.8 (130–336)
Intraoperative blood loss (ml)		34.1 ± 20.7
Intraoperative complications		0 (0)
Weight of excised gland(g)		174.5 ± 56.2 (76–275)
Volume of implants (cc)	Augmentation side	179.4 ± 26.5 (135–295)
	Reconstruction side	320.3 ± 56.7 (215–440)
Lymph node stage	N0	26 (78.8)
	N1	6 (18.2)
	N2	1 (3.0)
Stage	0	9 (27.3)
	I	11 (33.3)
	IIa	9 (27.3)
	IIb	3 (9.1)
	IIIa	1 (3.0)
ER	Positive	26 (78.8)
	Negative	7 (21.2)
PR	Positive	24 (72.7)
	Negative	9 (27.3)
HER2	Overexpression	6 (18.2)
	Negative	26 (78.8)
	Uncertain	1 (3.0)
Ki-67 (NA = 6)	≤ 30%	28 (84.8)
	> 30%	5 (1.5)
Chemotherapy	No	14 (42.4)
	Neoadjuvant	0 (0)
	Adjuvant	16 (48.5)
	Neoadjuvant and adjuvant	3 (9.1)
Radiotherapy	No	27 (81.8)
	Preoperative	0 (0)
	Postoperative	6 (18.2)
Hospital ward	24-h admission centre	18 (54.5)
	Inpatient unit	15 (45.5)
Length of hospital stay (days)		3.6 ± 3.3 (1–14)
Hospital cost (USD)		9132.1 ± 1389.1

*BMI* Body mass index, *NA* Not available, *ER* Oestrogen receptor, *PR* Progesterone receptor, *HER2* Human epithelial growth factor receptor type 2, *SLNB* Sentinel lymph node biopsy, *ALND* Axillary lymph node dissection

^a^The classification of breast ptosis is defined by Regnault[24] using the nipple position with respect to the inframammary fold

^b^Breast cup size is determined by the difference between the horizontal chest circumference at the level of the nipple and the horizontal chest circumference at the inframammary fold. The difference was ≤ 7.5 cm for the AA cup, ≤ 10 cm for the A cup, ≤ 12.5 cm for the B cup and ≤ 15 cm for the C cup

### Postoperative complications

No major complications occurred. Surgical complications occurred in a total of 6 patients (18.2%), including 3 patients (9.1%) with Clavien-Dindo grade I complications (1 case of haemorrhage treated with compression haemostasis and 2 cases of self-healing NAC ischaemia) and 3 patients (9.1%) with Clavien-Dindo grade II complications (3 cases of surgical site infection cured by oral antibiotics). In addition, 2 patients (6.1%) experienced cosmetic complications consisting of rippling and implant edge visibility. All complications occurred on the breast reconstruction side, and no complications occurred on the breast augmentation side. There were no cases of unplanned implant removal, unplanned return to the surgical theatre or unplanned hospital readmission for complications. During the median follow-up of 9.6 months, no cases of local recurrence or distant metastasis were observed. The complications are detailed in Table 2 and Additional file 4.

**Table 2:** Postoperative complications and oncological safety

		All patients *N* = 33 (%)
Clavien-Dindo classification		6 (18.2)
Grade I	Haemorrhage (compression haemostasis)	1 (3.0)
	NAC ischaemia (self-healing)	2 (6.1)
Grade II	SSI (oral antibiotics)	3 (9.1)
Grade ≥ III	Haemorrhage (surgical haemostasis)	0 (0)
	SSI (debridement)	0 (0)
	Wound dehiscence (reoperation)	0 (0)
	Skin flap necrosis (reoperation)	0 (0)
	NAC necrosis (reoperation)	0 (0)
	Others	0 (0)
Cosmetic complications	Rippling and implant edge visibility	2 (6.1)
	Others	0 (0)
Readmitted		0 (0)
Reoperated		0 (0)
Locoregional recurrence		0 (0)
Distant metastasis		0 (0)
Mortality		0 (0)

*NAC* Nipple-areola complex, *SSI* Surgical site infection

### Cosmetic results and quality of life

**Fig. 3:**
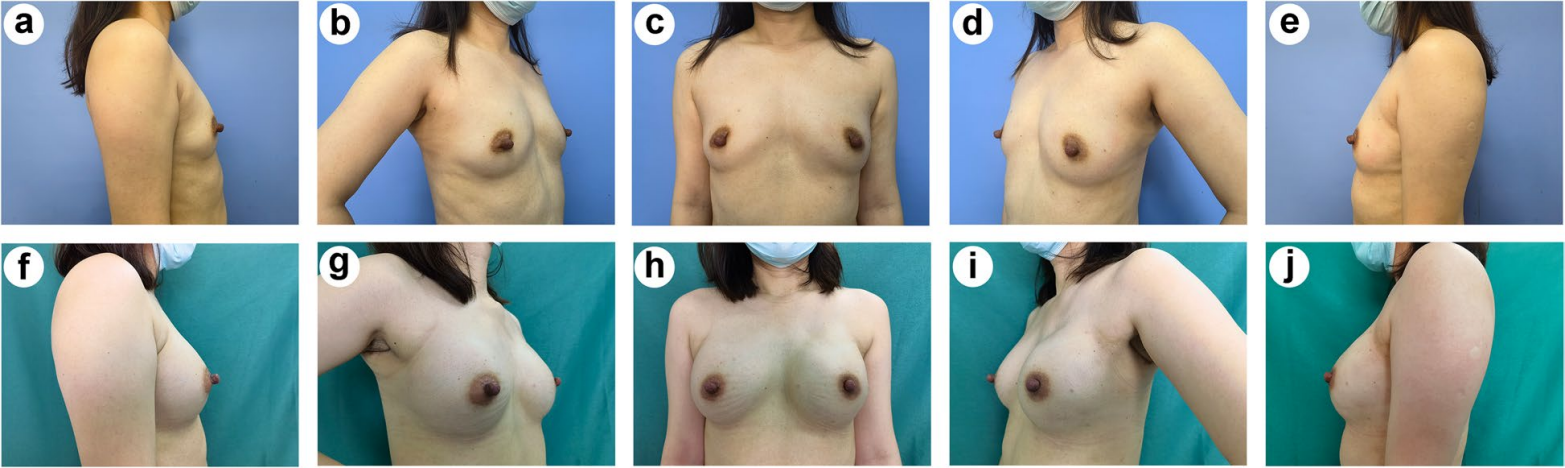
Representative case of an “Excellent” cosmetic outcome. **a**–**e** Preoperative images. **f**–**j** Three-month postoperative images

**Fig. 4:**
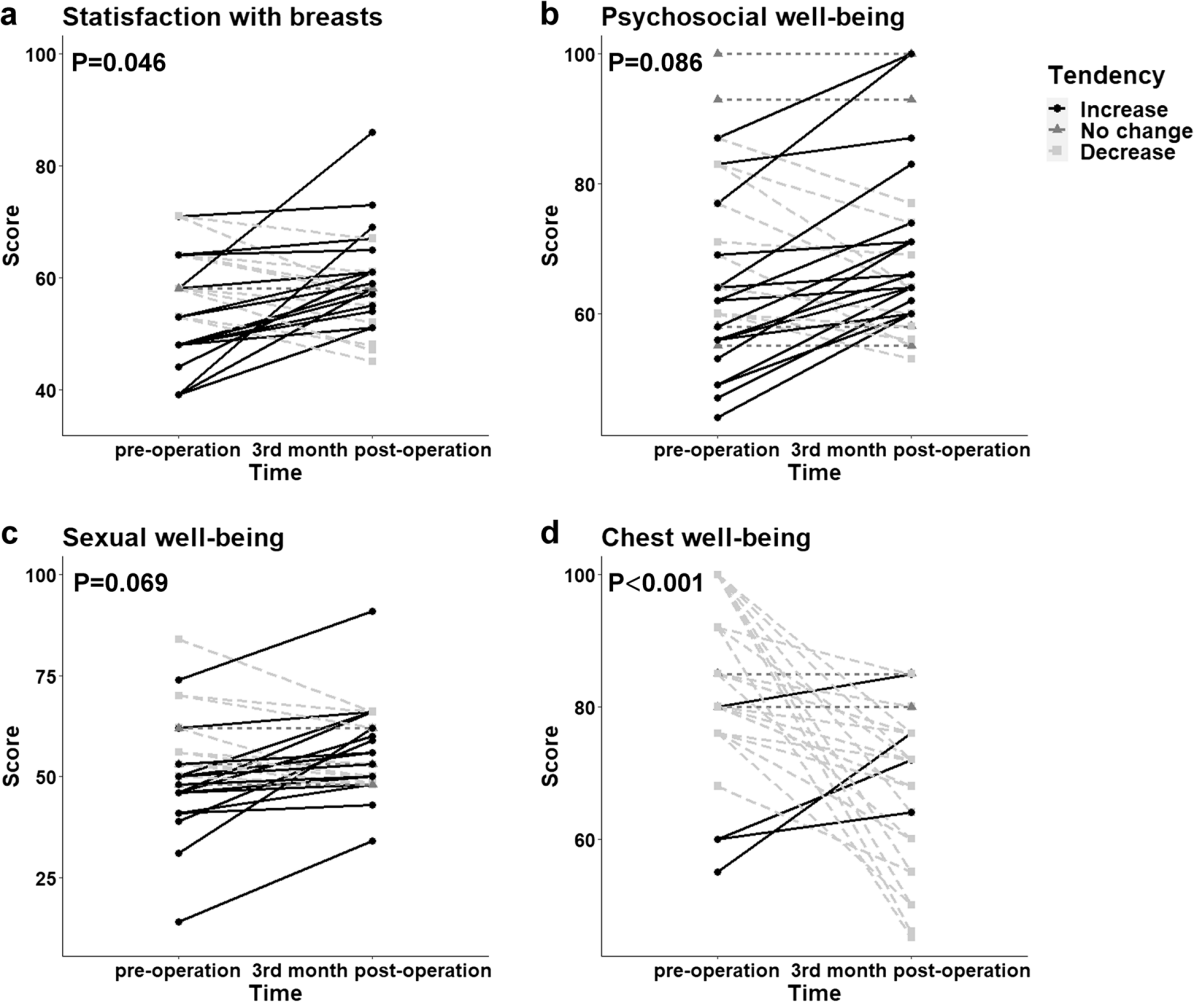
Preoperative and 3-month postoperative Breast-Q results

All the patients allowed their photographs (Additional file 5) to be taken and completed the Breast-Q (4 patients could not fill out the sexual well-being section) 3 months after surgery. In the doctor-reported cosmetic assessment, the outcome was rated as good or above for all patients, including 29 patients (87.9%) with an “Excellent” outcome and 4 patients (12.1%) with a “Good” outcome; a representative case is shown in Fig. 3. The patient-reported cosmetic assessment revealed a significant difference in breast satisfaction between the preoperative and 3-month postoperative scores (55.0 ± 9.5 vs. 58.8 ± 7.9, *P* = 0.046) (Fig. 4a).

In terms of the patient-based assessment of quality of life, there was no significant difference between the preoperative and 3-month postoperative scores for psychosocial well-being (67.2 ± 14.7 vs. 70.4 ± 13.9, *P* = 0.086) or sexual well-being (52.5 ± 13.8 vs. 55.7 ± 13.5, *P* = 0.069), but the average scores improved after surgery (Fig. 4b,4c); however, the score for chest well-being showed a significant decrease postoperatively (82.6 ± 11.8 vs. 69.3.2 ± 11.3, *P* < 0.001) (Fig. 4d).

## Discussion

In this study, for early breast cancer patients with small breasts (with no or mild-to-moderate breast ptosis), we proposed a novel endoscopic technique. i.e., direct-to-implant breast reconstruction and simultaneous contralateral breast augmentation (DTI-BR-SCBA), and prospectively analysed the outcomes in the first 33 surgical patients. The results showed that the surgical method yielded promising safety and cosmetic outcomes.

For patients with small breasts, BCS is not the ideal choice because of visible breast deformities after surgery [25], and breast implant-based reconstruction and contralateral breast augmentation can create more symmetrical breasts of ideal size and shape[26]. However, obvious breast incisions are inevitable in traditional operations, which not only influence the aesthetics (even IMF incisions cannot be hidden on a small breast) but also increase the risk of incision dehiscence or even implant loss under high skin tension. Therefore, traditional unilateral breast reconstruction with contralateral breast augmentation often requires two stages based on the surgeon's consideration for flap risk in DTI-BR and the opportunity for symmetry procedures in the second stage, which prolongs the treatment period and increases the patients’ physical and psychological burdens [27]. However, we can exempt the breast incisions through endoscopic technique to reduce the risk of incision complications and summarize the formula to select implants with appropriate size, thereby achieving DTI-BR with reliable safety and aesthetics. The patients can avoid the embarrassing period of breast asymmetry between the two surgeries and the trauma of the second surgery in two-staged operation and reduce surgical costs because delayed breast reconstruction is not covered by medical insurance in China.

In the previous endoscopic NSM procedure, the initial dissection plane was the dissection of the skin flap, followed by the retromammary space and subpectoral plane, and it was difficult to dissect the two inferior planes due to gravity and the superficial pressure of the gland after peeling off the mammary gland from the skin flap. However, when we reverse the dissection sequence, the gas tension formed by CO_2_insufflation would make the breast a tent-like structure, favouring the dissection of the subpectoral plane and the retromammary space. Even with rigid and relatively inflexible instruments, the whole operation process was smoother and more efficient owing to sufficient operating space and visual field. The use of “HUAXI Hole 1” addressed the difficulty of removing the mammary gland in the lower inner quadrant and the concern for inadequate tumour resection, and the surgical time was considerably reduced. In addition, the procedure did not require special surgical instruments, thus addressing the high cost of endoscopic and robotic-assisted breast surgery. Reportedly, the duration of open NSM with breast reconstruction is approximately 176 min [28], and the mean operative duration of endoscopic NSM is 250 min [29, 30]. In this study, the average total time required for our endoscopic DTI-BR-SCBA was 165.1 ± 36.1 min, including 50.7 ± 9.1 min for mastectomy and 22.2 ± 4.6 min for breast reconstruction, which is much shorter than that previously reported for unilateral endoscopic and even traditional breast reconstruction. The reduction of operation difficulty and surgery duration makes it possible to popularize the endoscopic surgery.

The outcomes of all patients were assessed as “Good” or “Excellent” by breast surgeons according to the Ueda scale and the patient-reported satisfaction with breasts was meaningfully enhanced at 3 months after the operation, indicating that the operation yielded good cosmetic outcomes due to the concealed scars and the improved appearance of the breasts. In terms of quality of life, more than half of the patients reported improved sexual and psychosocial well-being postoperatively, and the differences were borderline statistically significant. Reportedly, breast reconstruction following NSM significantly improves patient satisfaction [25, 31]. Our novel technique effectively reduces the incisions, thus further improving the cosmetic effects; thus, it is reasonable to expect that patients who undergo this procedure would have high satisfaction as they recover.

No intraoperative or major complication occurred in our study. The rate of surgical site infection was 9.1% in this study, which was quite lower than the 25% reported in a multicentre, prospective cohort study on mastectomy and immediate implant-based breast reconstruction in the UK [32]; thus, patients may benefit from the shortened operative duration and “no touch” endoscopic operation. The optimization of the endoscopic surgical field was conducive to reducing bleeding; only one case (3.0%) of haemorrhage occurred and was treated with compression haemostasis. In addition, the absence of incisions on the breast reduced the risk of skin flap and NAC necrosis, and there were no cases of flap necrosis and two cases (6.1%) of NAC ischaemia without complete nipple necrosis. There were no cases of implant loss, unplanned reoperation or unplanned readmission to the hospital for complications. Thus, the rates of all of these complications are in accordance with the UK National Quality Standards [32] (< 5% for reoperation, readmission, and implant loss and < 10% for infection).

However, there are also some limitations to this study. The number of patients was small, and the follow-up period was too short to monitor tumour safety and long-term cosmetic complications. We are actively increasing the sample size and further prolonging the follow-up duration. In addition, this was a single-arm study, and relevant comparative studies need to be designed for more accurate and complete results.

## Conclusions

In conclusion, it is suggested in this study that transaxillary endoscopic direct-to-implant breast reconstruction and simultaneous contralateral breast augmentation (DTI-BR-SCBA) may be a better alternative for early breast cancer patients with small breasts (with no or mild-to-moderate breast ptosis) because of its satisfactory postoperative safety and cosmetic outcomes and worthy of clinical promotion and application.

## Supplementary Information

**Additional file 1: **Contralateral endoscopic prepectoral breast augmentation.

**Additional file 2: **Endoscopic nipple-sparing mastectomy (followed by prepectoral breast reconstruction).

**Additional file 3: **Implant and mesh placement in endoscopic prepectoral breast reconstruction.

**Additional file 4: **Photos of postoperative complications (all occurred on the breast reconstruction side). (a) The recovery process of one patient with postoperative bleeding cured by compressing haemostasis. (b) The recovery process of one patient with infection cured by oral antibiotics. (c-d) Two cases of surgical site infection treated with oral antibiotics. (e) One case of self-healing NAC ischaemia necrosis with partial nipple volume loss. (f) One case of transient NAC ischaemia. (g-h) Two cases of rippling and visible implant edage. (c) and (g) are from the same patient.

**Additional file 5: **Photos of preoperative and 3-month postoperative breasts.

## Data Availability

The datasets used and/or analysed during the current study are available from the corresponding author on reasonable request.

## Abbreviations

DTI-BR-SCBA: Direct-to-implant breast reconstruction and simultaneous contralateral breast augmentation
BCS: Breast-conserving surgery
NSM: Nipple-sparing mastectomy
SSM: Skin-sparing mastectomy
DTI-BR: Direct-to-implant breast reconstruction
SLNB: Sentinel lymph node biopsy
ALND: Axillary lymph node dissection
IMF: Inframammary fold
NAC: Nipple-areolar complex
LDMF: Latissimus dorsi muscle flap
BMI: Body mass index
NA: Not available
ER: Oestrogen receptor
PR: Progesterone receptor
HER2: Human epithelial growth factor receptor type 2
SSI: Surgical site infection
