# Supplementary figures and images for: Short-term safety and cosmetic outcomes of endoscopic direct-to-implant breast reconstruction and simultaneous contralateral breast augmentation for breast cancer: a prospective analysis of 33 patients
Source: World J Surg Oncol. 2023 Jul 10;21:201. doi: 10.1186/s12957-023-03089-4 (PMC10332083; doi:10.1186/s12957-023-03089-4)

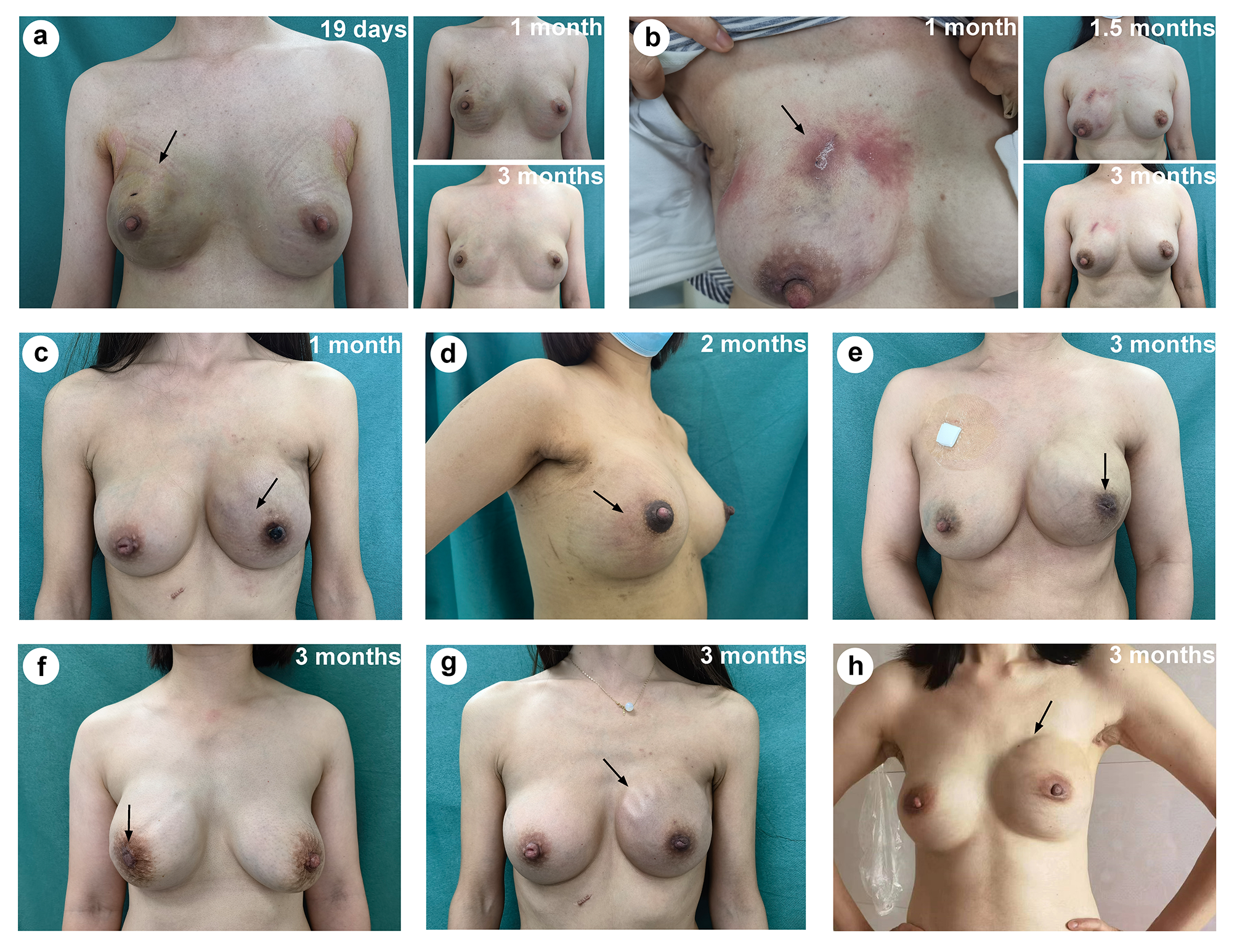

Supplement: Supplementary file 4 — Additional file 4: Photos of postoperative complications (all occurred on the breast reconstruction side). (a) The recovery process of one patient with postoperative bleeding cured by compressing haemostasis. (b) The recovery process of one patient with infection cured by oral antibiotics. (c-d) Two cases of surgical site infection treated with oral antibiotics. (e) One case of self-healing NAC ischaemia necrosis with partial nipple volume loss. (f) One case of transient NAC ischaemia. (g-h) Two cases of rippling and visible implant edage. (c) and (g) are from the same patient. [file 12957_2023_3089_MOESM4_ESM.png]
